# Gene knockdown of CCR3 reduces eosinophilic inflammation and the Th2 immune response by inhibiting the PI3K/AKT pathway in allergic rhinitis mice

**DOI:** 10.1038/s41598-022-09467-4

**Published:** 2022-03-30

**Authors:** Jiasheng Yuan, Yuehui Liu, Juan Yu, Meina Dai, Yu Zhu, Youwei Bao, Haisen Peng, Ke Liu, Xinhua Zhu

**Affiliations:** Department of Otolaryngology-Head and Neck Surgery, The Second Affiliated Hospital of Nanchang University, Nanchang, 330006 China

**Keywords:** Inflammation, Respiratory tract diseases, Pathogenesis

## Abstract

The CCR3 gene plays a critical role in allergic airway inflammation, such as allergic rhinitis (AR), and there is an inflammatory signal link between the nasal cavity and the CCR3 gene in bone marrow. However, the effects of the CCR3 gene in bone marrow cells on AR are not clear. The present study investigated the roles and underlying mechanisms of the bone marrow CCR3 gene in AR mice. Conditional knockout of the bone marrow CCR3 gene (CKO) in mice was generated using the Cre-LoxP recombination system, and offspring genotypes were identified using polymerase chain reaction (PCR). An ovalbumin-induced AR model was established in CKO and wild-type mice to measure eosinophilic inflammation and the Th2 immune response. The following mechanisms were explored using a specific PI3K/AKT pathway inhibitor (Ly294002). We successfully constructed and bred homozygous CKO mice and confirmed a significant increase in CCR3 expression and PI3K/AKT pathway activity in AR mice. Deficiency of the bone marrow CCR3 gene caused a remarkable reduction of CCR3 expression and the PI3K/AKT signaling pathway activity, inhibited histopathological lesions and eosinophil infiltration of the nasal cavity, and reduced the production of Th2 cytokines in serum, which led to the remission of allergic symptoms in AR mice. Ly294002 treatment also decreased these inflammatory indexes in a concentration-dependent manner and blocked inflammatory signals from CCR3, but it did not affect the high expression of CCR3 in AR mice. Collectively, our results suggest that conditional knockout of the bone marrow CCR3 gene can reduce eosinophilic inflammation and the Th2 immune response, which may be due to inhibition of the PI3K/AKT pathway.

## Introduction

Allergic rhinitis (AR) is a non-infectious disease of the nasal mucosa in atopic individuals^1^. The accumulation of eosinophils (Eos) in the nasal mucosa and Th1/Th2 immune imbalance are key features of AR^1–3^. Dendritic cells present antigens in the early sensitization stage and promote Th2 cells to secrete numerous Th2 cytokines, such as interleukin (IL)-4, IL-5 and IL-13. Activated Eos infiltrate the nose in the late challenge stage and release inflammatory mediators that produce local tissue damage and allergic symptoms^1,3,4^. However, the mechanisms of AR are not fully understood due to the complex processes of Eos migration and Th1/Th2 immune imbalance.

C–C chemokine receptor 3 (CCR3) is a G protein-coupled membrane receptor that is predominantly expressed on Eos^5,6^. Eos are the main effector cells in the pathogenesis of AR. After specific activation of eotaxin, the CCR3 receptor on the surface of Eos triggers the recruitment of a large number of eosinophils to the nose. Activated Eos exert inflammatory effects by releasing a variety of inflammatory mediators locally, which produce cytotoxic effects, such as tissue edema and mucosal destruction^1,4,7–9^. CCR3 is also expressed on Th2 cells, and it is involved in the differentiation of Th2 cells and the secretion of Th2 cytokines^5,10^. Our previous studies^11^ found that down-regulating the expression of CCR3 using RNA interference prevented the nasal infiltration and degranulation of Eos in AR mice. The pro-inflammatory functions of mast cells in vivo were also inhibited after the inhibition of the CCR3 gene^12^. These cells are tightly associated with allergic airway diseases^1^, which suggests that the CCR3 gene is an important regulator of allergic inflammation. However, the effects of targeted knockout of bone marrow CCR3 gene on eosinophilic inflammation in AR mice are unclear.

Phosphatidylinositol-3-kinase (PI3K) and serine/threonine protein kinase (AKT) act as downstream signal effectors of G protein-coupled receptors and jointly form the intracellular PI3K/AKT signaling pathway^13^. The PI3K/AKT pathway contributes to allergic airway inflammation by promoting eosinophilic recruitment and Th1/Th2 immune imbalance^13–16^. PI3K/AKT pathway activity is increased in asthmatic mice. However, asthmatic mice treated with PI3K-specific inhibitors showed a reduction in Th2 cytokine production, the infiltration of pulmonary eosinophils and the degree of airway inflammation^15,17,18^. There may be a molecular connection between membrane surface CCR3 and the intracellular PI3K/AKT pathway^19,20^. Saito^20^ reported that PI3Kγ was a key molecule in allergic airway inflammation. The inhibition of PI3Kγ attenuated Eos functions by suppressing the downstream signal of CCR3^20^. Our previous in vitro experiments found that conditional knockout of the bone marrow CCR3 gene effectively inhibited the proliferation, migration and degranulation of bone marrow-derived Eos via inhibition of the PI3K/AKT signaling pathway. However, whether the PI3K/AKT pathway is involved in the pathogenesis and whether CCR3 regulates allergic inflammation by targeting the PI3K/AKT pathway in AR are not known.

Therefore, the present study investigated the effects and mechanisms of the bone marrow CCR3 gene in the pathogenesis of AR at the animal level using conditional knockout.

## Results

### Genotypic identification of offspring mice

To obtain homozygous mice with conditional knockout of the bone marrow CCR3 gene (CKO), we propagated CKO mice and identified the genotypes of their offspring using polymerase chain reaction (PCR) and agarose gel electrophoresis (see Supplementary Fig. S1 online). Obvious bands were found at 591 bp in the PCR swimming lane of the CCR3-Loxp gene (Supplementary Fig. S1a) on No. 321–342 and at 750 bp in the PCR swimming lane of the Lyz2-Cre gene (Supplementary Fig. S1b) on No. 322, 324–325, 327–329, 331–334, 336–340, and 342. Therefore, mice containing the CCR3-Loxp and Lyz2-cre genes were identified as CKO homozygous mice for use in subsequent experiments (No. 322, 324–325, 327–329, 331–334, 336–340, and 342).

### Gene knockdown of CCR3 alleviates nasal symptoms in AR mice

**Figure 1:**
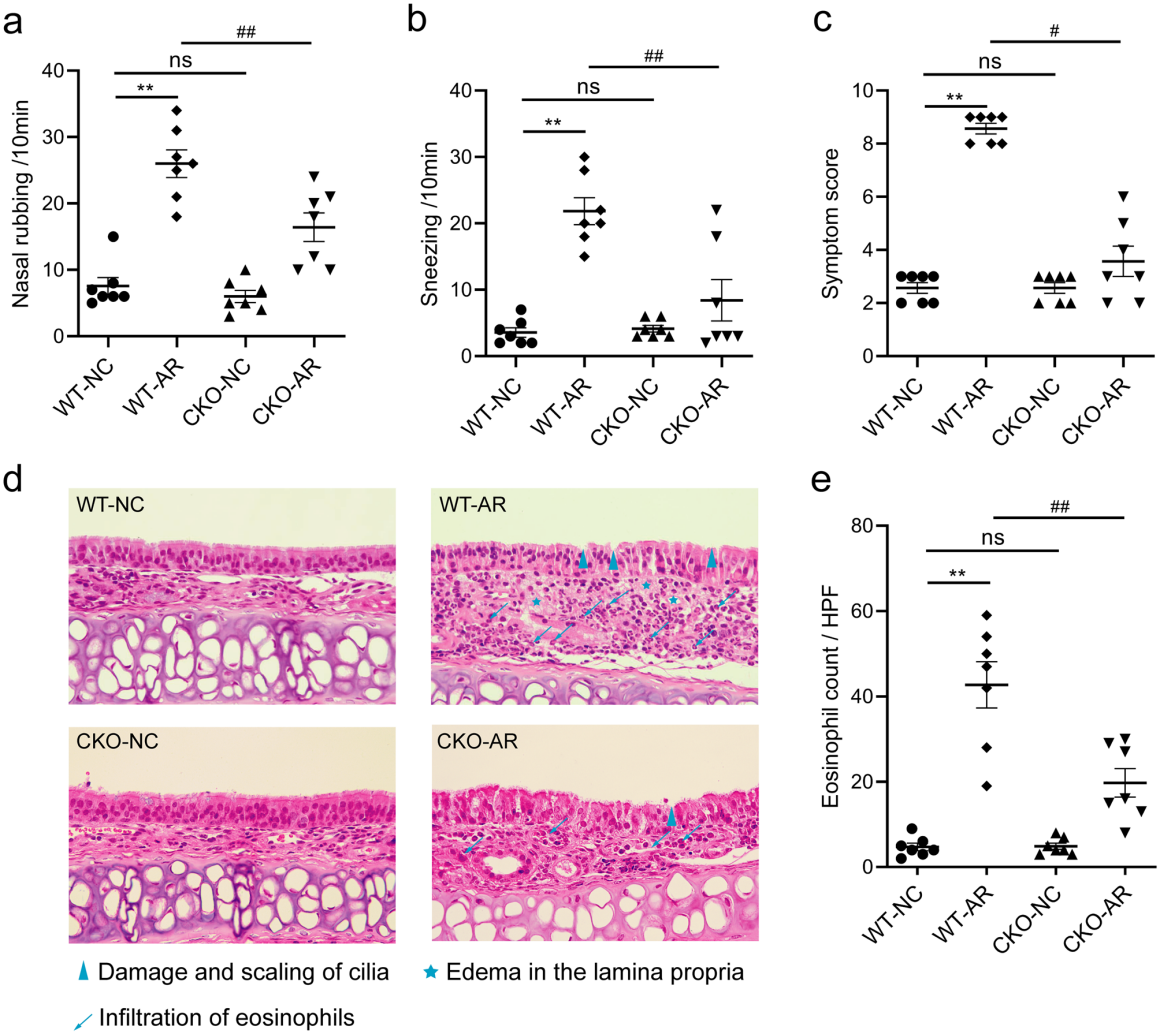
Effects of CCR3^−/−^ (CKO) on behavioral performance and histological morphology. The times of nasal rubbing (**a**), sneezing (**b**) and symptom score (**c**) within 10 min after the final challenge. HE staining images (× 400 magnifications) (**d**). Eosinophil count in nasal septal mucosa (**e**). *WT-NC* wild-type control group, *WT-AR* wild-type AR group, *CKO-NC* CCR3^−/−^ (CKO) control group, *CKO-AR* CKO AR group. Data are expressed as the means ± SEM. **P < 0.01 vs. the WT-NC group; ^#^*P* < 0.05, ^##^*P* < 0.01 vs. the WT-AR group, ns indicates* P* > 0.05.

To examine the effects of the CCR3 gene on nasal allergic symptoms in AR mice, we evaluated the behavioral performance of mice in each group (Fig. 1a–c). Compared to the wild-type control (WT-NC) group, mice in the wild-type AR (WT-AR) group had obvious AR symptoms, such as nasal rubbing and sneezing, and the symptom score was greater than 5 points, which indicated successful construction of the ovalbumin (OVA)-induced AR mice. CCR3 gene-deficient mice exhibited no significant differences in nasal symptoms or symptom scores between the CKO control (CKO-NC) group and the WT-NC group. However, mice in the CKO AR (CKO-AR) group exhibited significantly lower nasal symptoms and symptom scores compared to the WT-AR group, which suggested that targeted knockout of the bone marrow CCR3 gene alleviated the nasal allergic symptoms of OVA-induced AR mice.

### Gene knockdown of CCR3 reduces pathological damage to the nasal mucosa in AR mice

To investigate the role of the CCR3 gene in nasal inflammation, we observed the histopathology of the nasal mucosa after HE staining (Fig. 1d) and counted the number of Eos in high magnification fields (Fig. 1e). No obvious abnormalities were observed in the nasal mucosa of mice in the WT-NC group or the CKO-NC group. In contrast, mice in the WT-AR group exhibited obvious disorder of the nasal mucosa. A large number of cilia were damaged and shedding, and the lamina propria of the mucosa exhibited obvious congestion, swelling and a massive infiltration of Eos. However, these nasal pathological lesions and Eos infiltration were significantly improved in the CKO-AR group mice, which indicated that targeted knockdown of the CCR3 gene reduced local mucosal damage and Eos infiltration in AR mice.

### Gene knockdown of CCR3 regulates serum cytokine levels in AR mice

**Figure 2:**
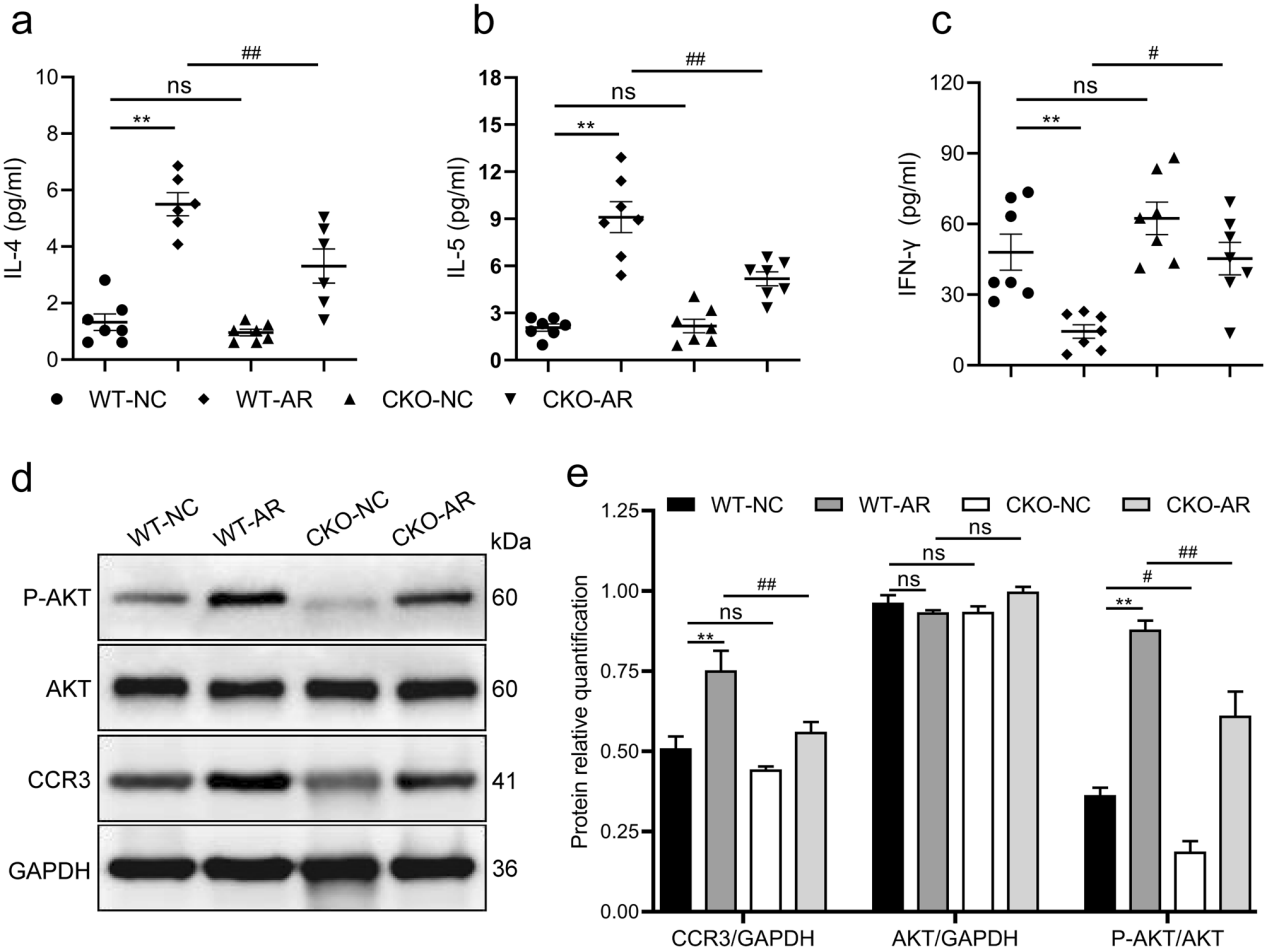
Effects of CCR3^−/−^ (CKO) on the levels of Th1 and Th2 cytokines and the protein contents of CCR3, AKT and P-AKT. The levels of IL-4 (**a**), IL-5 (**b**) and IFN-γ (**c**) in serum using ELISA. Representative Western blot images (**d**). Relative protein quantifications of CCR3, AKT and P-AKT in the nasal mucosa (**e**). Data are expressed as the means ± SEM. **P < 0.01 vs. the WT-NC group; ^#^*P* < 0.05, ^##^*P* < 0.01 vs. the WT-AR group, ns indicates* P* > 0.05.

To investigate whether the CCR3 gene was associated with Th1/Th2 immunity, we detected the serum levels of Th2 (IL-4 and IL-5) and Th1 (IFN-γ) cytokines using ELISA. As depicted in Fig. 2a–c, the serum levels of Th2 cytokines increased considerably in the WT-AR group, and the level of Th1 cytokines decreased. In contrast, the concentration of Th2 cytokines in serum decreased in the CKO-AR group, and the concentration of Th1 cytokines increased. These results indicate that AR is an allergic inflammation dominated by a Th2-type immune response, and targeted knockdown of the CCR3 gene reverses the immune imbalance of Th1/Th2 cytokines in AR mice.

### Gene knockdown of CCR3 decreases the levels of CCR3 protein and PI3K/AKT pathway-phosphorylated protein in the nasal mucosa of AR mice

We detected the protein expression of CCR3, AKT and phosphorylated AKT (P-AKT) in the nasal mucosa of mice using Western blotting (Fig. 2d,e). The protein levels of CCR3 in the nasal mucosa of the WT-AR group were remarkably higher than the WT-NC group, and the expression levels of CCR3 in the CKO-AR group were significant lower than the WT-AR group, which revealed that targeted knockdown of the CCR3 gene effectively reduced CCR3 protein levels. Notably, we also found that the level of P-AKT protein, which reflects the degree of PI3K/AKT pathway activation, was significantly increased in the nasal mucosa of AR mice. Conversely, the protein expression of P-AKT in the CKO-AR group was markedly down-regulated. There was no significant difference in the total AKT protein level of each group. These preliminary observations demonstrated that the PI3K/AKT pathway was aberrantly activated in AR mice, and could be inhibited by targeted knockdown of the CCR3 gene. Our preliminary pre-experiments showed that targeted knockout of the bone marrow CCR3 gene inhibited the proliferation and chemotaxis of Eos in vitro via inhibition of the PI3K/AKT pathway. However, whether the PI3K/AKT pathway was involved in the regulatory mechanism of AR was not clear. Therefore, more in-depth experiments were performed to explore this scientific question.

### Ly294002 concentration-dependently relieves nasal symptoms in AR mice

**Figure 3:**
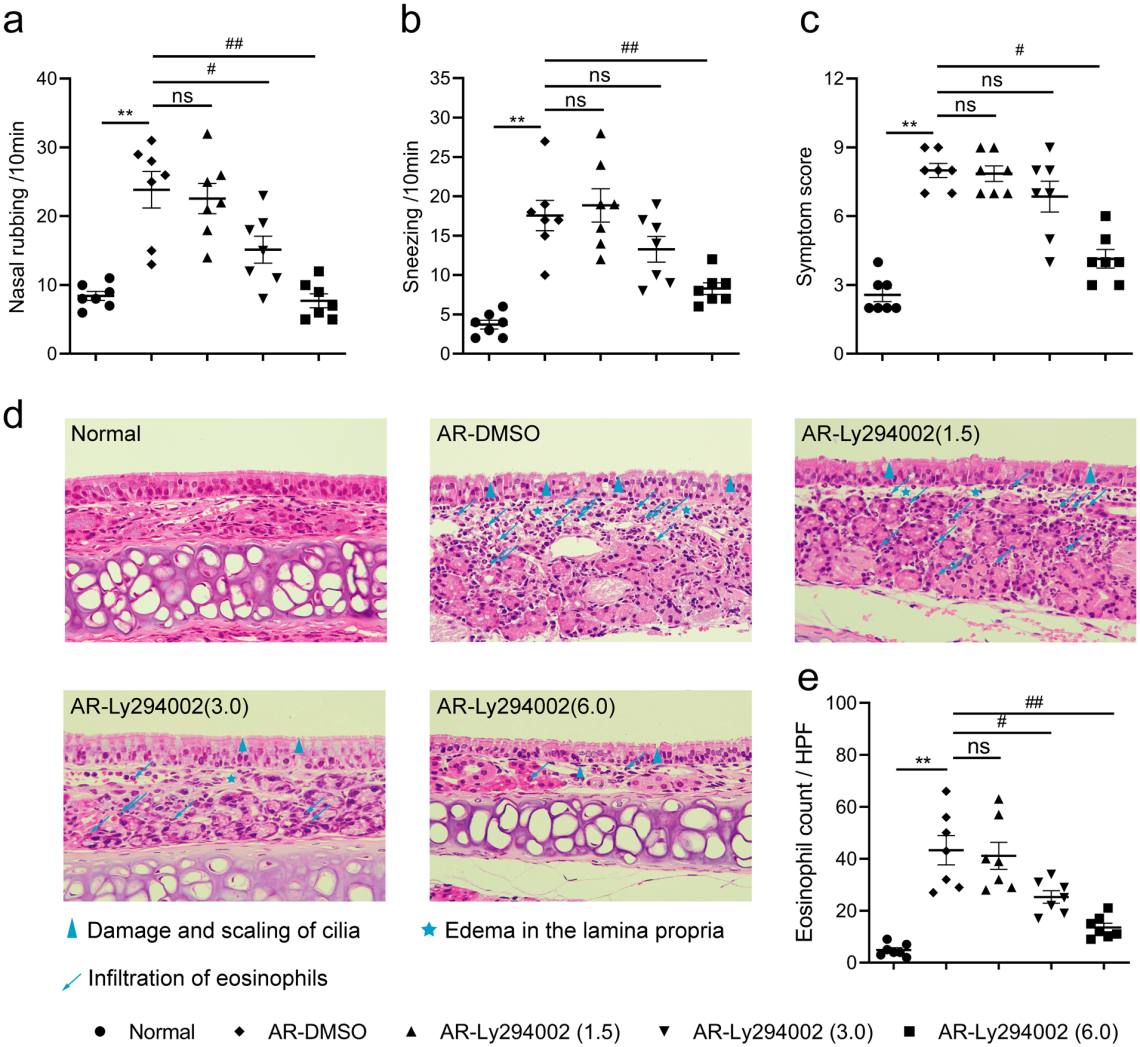
Effects of Ly294002 on behavioral performance and histological morphology. The times of nasal rubbing (**a**), sneezing (**b**) and symptom score (**c**) within 10 min after the final challenge. HE staining images (× 400 magnifications) (**d**). Eosinophil counts in nasal septal mucosa (**e**). DMSO: the solvent. Ly294002: specific PI3K/AKT inhibitor. Data are expressed as the means ± SEM. **P < 0.01 vs. the Normal group; ^#^*P* < 0.05, ^##^*P* < 0.01 vs. the AR-DMSO group, ns indicates *P* > 0.05.

To further determine whether the PI3K/AKT pathway was directly implicated in the pathogenesis of AR, different concentrations (0–6.0 mg/kg) of the PI3K/AKT pathway-specific inhibitor Ly294002 were intraperitoneally injected into AR mice one hour before each challenge. We assessed the nasal symptoms of mice within 10 min after the last challenge (Fig. 3a–c). Similar to the WT-AR group, the AR solvent control (AR-DMSO) group showed obvious nasal symptoms, and the symptom score was greater than 5. However, the allergic symptoms of AR mice gradually improved with increasing concentrations of Ly294002 in the PI3K inhibitor groups. These results demonstrated that a highly selective inhibitor of the PI3K/AKT pathway, Ly294002, concentration-dependently relieved nasal symptoms in AR mice.

### Ly294002 attenuates pathological damage to the nasal mucosa in AR mice

To investigate the potential role of the PI3K/AKT pathway on nasal inflammation, we evaluated the influence of different concentrations of Ly294002 on the histology of nasal mucosa using HE staining. As shown in Fig. 3d,e, the nasal mucosa of AR-DMSO group mice showed obvious damage and Eos infiltration. However, these changes were progressively ameliorated in AR mice with increasing concentrations of Ly294002. These results suggested that the PI3K/AKT pathway promoted pathological damage and Eos infiltration in the nasal mucosa of AR mice, and Ly294002 inhibited this pathophysiological process.

### Ly294002 modulates serum cytokine levels in AR mice

**Figure 4:**
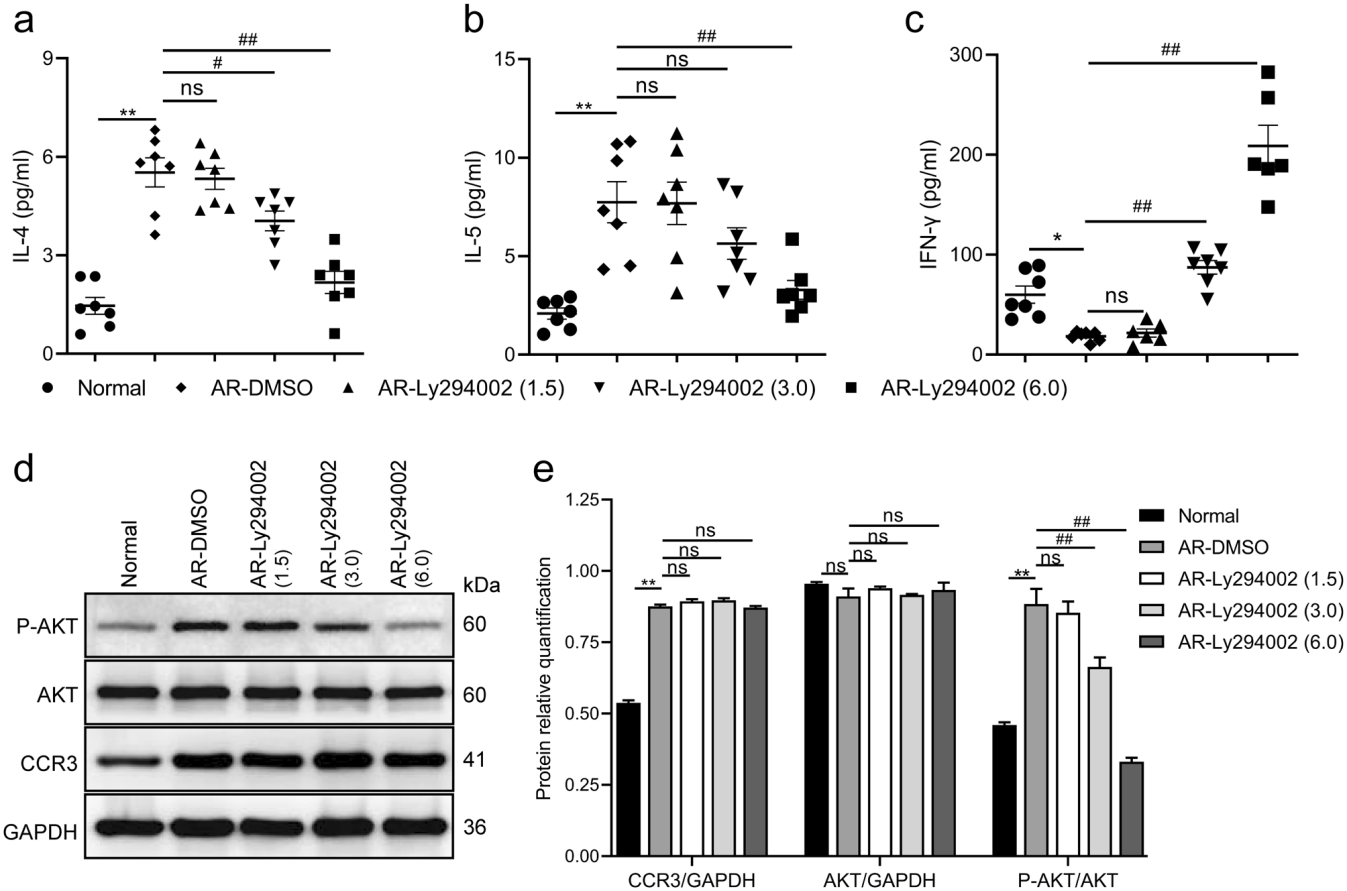
Effects of Ly294002 on the levels of Th1 and Th2 cytokines and the protein contents of CCR3, AKT and P-AKT. The levels of IL-4 (**a**), IL-5 (**b**) and IFN-γ (**c**) in serum using ELISA. Representative Western blot images (**d**). Relative protein quantifications of CCR3, AKT and P-AKT in the nasal mucosa (**e**). Data are expressed as the means ± SEM. *P < 0.05, **P < 0.01 vs. the normal group; ^#^*P* < 0.05, ^##^*P* < 0.01 vs. the AR-DMSO group, ns indicates *P* > 0.05.

To investigate the role of the PI3K/AKT pathway on Th1/Th2 immunity in AR mice, we detected the serum levels of Th1/Th2 cytokines using ELISA (Fig. 4a–c). An imbalance of Th1/Th2 cytokines exists in AR mice, but Ly294002 treatment corrected this immune imbalance. The degree of correction of the Th1/Th2 immune imbalance was proportional to the concentration of Ly294002. These results showed that Ly294002 treatment gradually reduced the level of Th2 cytokines and increased the level of Th1 cytokines in serum to reverse the imbalance of Th1/Th2 cytokines in AR mice.

### Ly294002 decreases the levels of PI3K/AKT pathway-phosphorylated protein, but not CCR3 protein, in the nasal mucosa of AR mice

We detected the protein levels of CCR3, AKT and P-AKT in the nasal mucosa using Western blotting. As indicated in Fig. 4d,e, Ly294002 decreased P-AKT levels in the nasal mucosa of AR mice in a concentration-dependent manner but did not change the total AKT level. This result demonstrated that intraperitoneal injection of a high concentration of Ly294002 effectively suppressed activation of the PI3K/AKT pathway. Notably, the high expression of CCR3 in the nasal mucosa of AR mice was not inhibited even at a high concentration. These results confirmed that the PI3K/AKT pathway was involved in the pathogenesis of AR. Specific inhibition of the PI3K/AKT pathway reduced the degree of inflammation in AR mice but did not influence expression of the CCR3 protein.

## Discussion

The present study successfully constructed a murine model of allergic rhinitis (AR) using ovalbumin (OVA) for systemic sensitization and local excitation. We demonstrated that AR mice were in a state of Th1/Th2 immune imbalance, and the infiltrated eosinophils (Eos) damaged the structure of the nasal mucosa and induced allergic symptoms.

The recruitment, activation and degranulation of Eos from the bone marrow to the nasal cavity are the key factors of pathological damage to the nasal mucosa in AR mice, and Eos are the main cells expressing CCR3^1,4,21^. To examine whether targeted knockout of the bone marrow CCR3 gene would impact eosinophilic inflammation and the Th2 immune response in AR mice, wild-type (WT) mice and previously constructed conditional knockout bone marrow CCR3 genotype (CKO) mice were used for experiments. The results showed that targeted knockout of the bone marrow CCR3 gene efficiently inhibited eosinophilic infiltration, pathological damage in nasal mucosa and Th2 cytokine levels in serum, which alleviated the allergic symptoms of AR mice. The deletion of CCR3 gene in bone marrow blocked the connection between eotaxin and CCR3 receptor on the surface of Eos, which may be the major reason for the reduction of pathology and symptoms in CKO-AR mice. Although the nasal pathology and nasal allergy symptoms of AR mice were largely alleviated, these changes were not completely alleviated after CCR3 down-regulation. This result may be due to receptors other than CCR3, such as CCR4^22^ and Toll-like receptors^23^, which are also involved in the activation of Eos, and deletion of CCR3 cannot completely inhibit the biological effects of other inflammatory cells.

Theoretically, targeted knockout of the bone marrow CCR3 gene refers to knockout of the CCR3 gene in all cells in bone marrow^24^. In addition to being mainly expressed in Eos^9^, CCR3 is also expressed in mast cells^12^ and CD4^+^ T cells^25^, which cooperate to promote the pathogenesis of AR. Our previous studies^12,26^ confirmed that the lentivirus-mediated CCR3 interference vector inhibited the proliferation, migration and degranulation of mast cells in vitro and in vivo, which ameliorated AR in a murine model. The serum Th1 and Th2 cytokine results showed that down-regulation of the CCR3 gene reduced Th2 immunity of AR mice, but the function of CD4^+^ T cells must be further explored to explain the detailed mechanism of this phenomenon.

Notably, we also found that targeted knockdown of the CCR3 gene reduced the protein levels of CCR3 and P-AKT, which reflects the activity of the PI3K/AKT pathway in nasal mucosa. Previous studies^13–16^ established that abnormal activation of the PI3K/AKT signaling pathway was an important pathophysiological mechanism of allergic diseases. The αi subunit of G protein is activated when eotaxin binds to the CCR3 receptor on the surface of Eos, and PI3K is activated by the binding of the β-γ subunit of GTP binding protein, which activates the downstream AKT signaling pathway^19^. These results suggest a structural basis of the signaling link between the surface membrane CCR3 receptor on the surface of Eos and the intracellular PI3K/AKT pathway. To further examine the influence of the PI3K/AKT pathway on the pathogenesis of AR and its underlying molecular mechanisms, we used the specific PI3K inhibitor Ly294002.

Ly294002 is a synthetic compound and specific inhibitor of PI3K in airway inflammation, tumors and immune diseases^27–29^. Ly294002 treatment effectively attenuates airway inflammation by reducing airway hyperresponsiveness^30^, airway remodeling^29,31^ and eosinophilic infiltration^32^ in asthmatic mice. However, the association of the PI3K/AKT pathway with AR was not fully elucidated. We treated AR mice with different concentrations of Ly294002. Intraperitoneal injection of Ly294002 decreased the number of Eos in nasal mucosa and the contents of Th2 cytokines in serum in a concentration-dependent manner and progressively alleviated the nasal symptoms of AR mice. There is signal crosstalk between the PI3K pathway and the Ras-ERK pathway^33^, which may also be involved in the pathogenesis of AR^19^. However, whether inhibition of the PI3K pathway leads to compensatory activation of the ERK pathway is not clear and provides a new direction for further research.

CCR3 is a G-protein-coupled receptor on the surface of the eosinophil membrane^1^, and PI3K/AKT is an intracellular signaling pathway downstream of the G-protein-coupled receptor^13^. Targeted knockdown of the CCR3 gene decreased the levels of CCR3 protein and suppressed activation of the PI3K/AKT pathway in Eos, which suggested that the down-regulation of the CCR3 gene affected PI3K/AKT signaling in Eos of AR mice. Our preliminary in vitro experiments also found that targeted knockout of the bone marrow CCR3 gene down-regulated the expression of the PI3K/AKT signaling pathway in Eos and inhibited the proliferation, migration and degranulation of eosinophils in vitro. However, Ly294002 inhibited activation of the PI3K/AKT signaling pathway, but it failed to suppress the high expression of CCR3 protein. These results further suggest that Ly294002 inhibits the inflammatory signal from upstream CCR3 in AR mice by blocking activation of the PI3K/AKT pathway in Eos.

Taken together, the present study confirmed that specific blockade of bone marrow CCR3 alleviated AR in mice by affecting the migration of Eos from bone marrow to the nasal mucosa and alleviating the Th2 immune response. We further revealed that bone marrow CCR3 knockout ameliorated eosinophilic inflammation via inhibition of the downstream PI3K/AKT signaling pathway of the G protein-coupled receptor, which provides a reference for more accurate targeted therapy.

## Materials and methods

### Animals

Six to eight-week-old male C57BL/6 mice (20–25 g) were used in the experiments. All animals were housed in the SPF experimental animal room with independent access to aseptic water and aseptic feed. The Medical Research Ethics Committee of The Second Affiliated Hospital of Nanchang University approved the present study (No. 2020-A701). All experiments were performed in accordance with relevant named guidelines, regulations and the ARRIVE guidelines.

### Construction strategy of conditional knockout of bone marrow CCR3 genotype (CKO) mice

The male C57BL/6 mice of CKO were constructed with assistance from the Nanjing Biomedical Research Institute of Nanjing University (NBRI) using the Cre-LoxP recombinase system. The principle^24^ was that two LoxP sites were inserted on both sides of the CCR3 gene coding exon in mice via gene targeting, and then hybridized with mice expressing Cre enzyme in bone marrow cells to obtain mice in which the target gene of bone marrow CCR3 was specifically knocked out.

**Figure 5:**
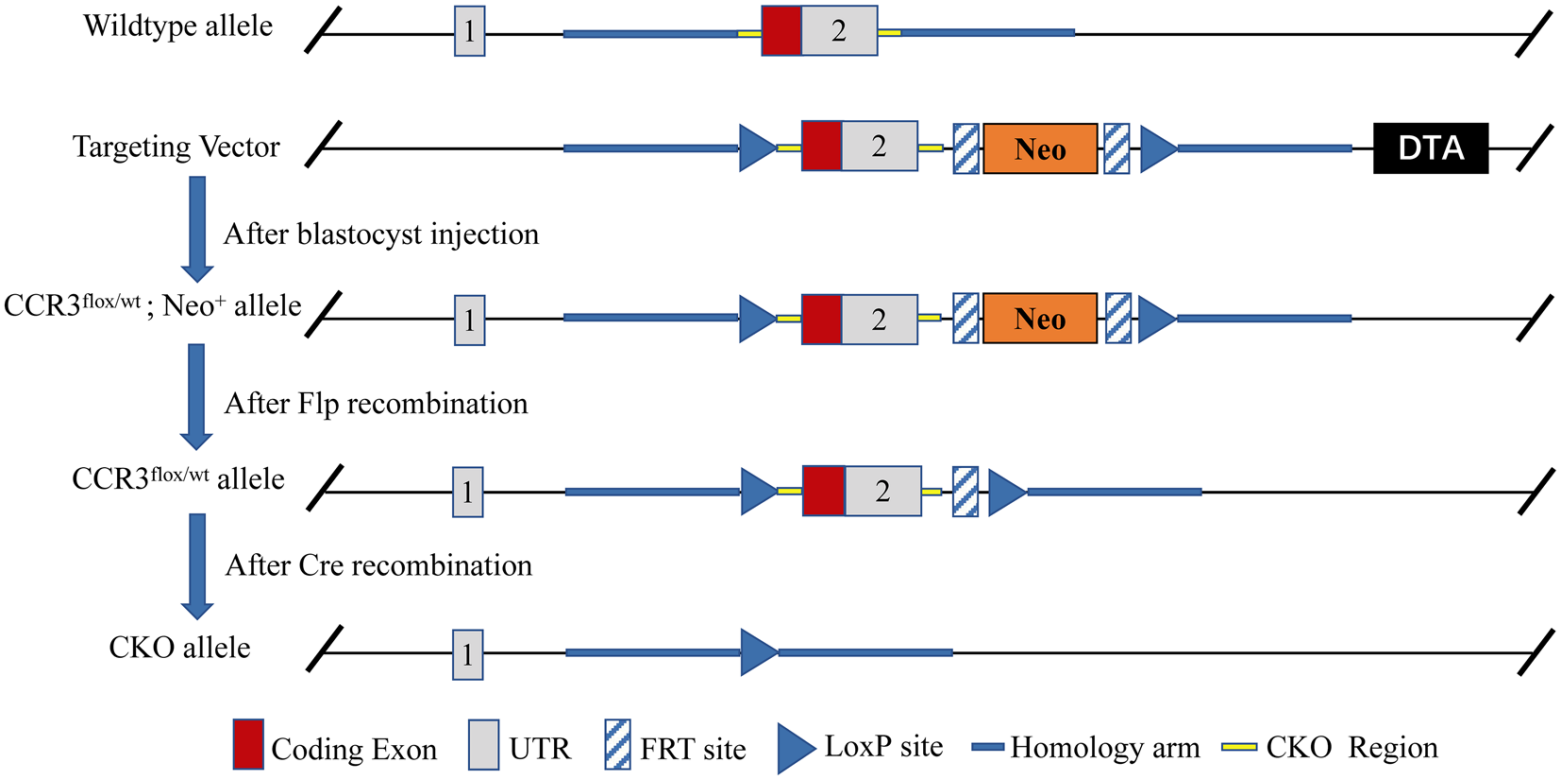
Construction strategy for the CCR3^−/−^ (CKO) mice.

As shown in Fig. 5, gene targeting technology was used to construct a CCR3 gene targeting vector, which carried a neomycin-resistance gene (Neo) cassette flanked by Flippase recognition target (FRT) sites and two LoxP sites inserted on both sides of the coding exon. The targeting vector was linearized and electroporated into C57BL/6 embryonic stem (ES) cells, and positive ES cells were screened by identifying positive and negative genes on the vector. Microinjection was used to inject a certain quantity of positive ES cells into mouse blastocysts, which were transferred into the uteri of pseudo-pregnant mice to obtain chimeric mice (CCR3^flox/wt^; Noe^+^). The chimeric mice were mated with mice expressing Flippase (FLP) all over the body to produce CCR3^flox/wt^ mice in which the Neo gene was removed. The CCR3^flox/wt^ mice were crossed with B6.129P2-Lyz2^tm1(cre)Ifo^/NJU mice (NBRI, Nanjing, China) that specifically expressed Lyz2-Cre recombinase in bone marrow cells, and heterozygous mice (CCR3^flox/wt^; Cre^+^) with conditional knockout of the CCR3 gene in bone marrow were successfully constructed. The CCR3^flox/wt^; Cre^+^ mice were crossed between each other to generate homozygous CKO offspring (CCR3^flox/flox^; Cre^+^).

### PCR primers and genotyping

Genomic DNA was isolated from mouse tails for PCR genotyping as described previously^34^. Briefly, genomic DNA was extracted using the alkaline lysis method and amplified using PCR amplification. The PCR amplification products were detected using agarose gel electrophoresis for DNA identification. 5′-GAGAAGCTCTTGGGATATCAATGC-3′ and 5′-GAAGGGGGGAATTCCAAATTTT-3′ are the sequences of the PCR primers used for the CCR3-LoxP allele. 5′-CCCAGAAATGCCAGATTACG-3′ and 5′-CTTGGGCTGCCAGAATTTCTC-3′ were the primers for the Lyz2-Cre allele.

### AR model and Ly294002 treatment

**Figure 6:**
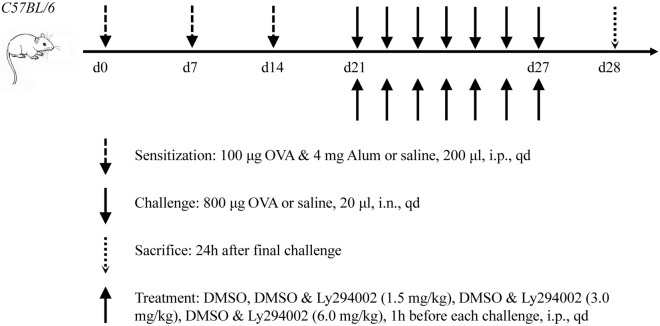
Schematic diagram of the AR model and treatment with Ly294002. *i.p*. intraperitoneal injection, *i.n*. intranasal administration, *qd* quaque die.

An ovalbumin (OVA)-induced AR mouse model was established (Fig. 6)^25^. The CKO mice and some wild-type (WT) mice were randomly divided into 4 groups (n = 7 per group). Mice in the AR groups (WT-AR group and CKO-AR group) were sensitized via a 200 μl intraperitoneal injection of 100 μg OVA (Sigma-Aldrich, St. Louis, USA) and 4 mg aluminum hydroxide (Thermo Fisher Scientific, Waltham, MA, USA) on days 0, 7 and 14, and challenged once daily with a 20 μl intranasal administration of 800 μg OVA on days 21–27. Blank control mice (WT-NC group and CKO-NC group) were treated with saline instead of OVA. Twenty-four hours after the last challenge, the mice were sacrificed using anesthetics, and the specimens were collected.

Another group of WT mice was randomly divided into 5 groups (n = 7 per group). One hour before each intra-nasal challenge, mice in the PI3K inhibitor groups (AR-DMSO group, AR-Ly294002(1.5) group, AR-Ly294002(3.0) group and AR-Ly294002(6.0) group) were intraperitoneally injected with different concentrations of Ly294002 (MedChemExpress, New Jersey, USA) mixtures containing 2% DMSO. On the basis of AR model construction, the injected concentrations of Ly294002 in these groups were 0 mg/kg, 1.5 mg/kg, 3.0 mg/kg and 6.0 mg/kg. The Normal mouse group was treated with saline instead of Ly294002 mixtures.

### Evaluation of nasal symptoms

Within 10 min after the final challenge, nasal rubbing and sneezing were recorded, and a symptom score was calculated as described previously^35^. Briefly, effective nasal rubbing was recorded when the mice scratched their nose with both front claws. Valid sneezing was recorded when the mice made the sound of "an choo" accompanied by head shaking. Nasal secretions were observed at the time of the deadline, and the symptoms were scored according to the standards listed in Supplementary Table S1. The AR model was considered successfully constructed when the total score was greater than 5.

### Hematoxylin and eosin (HE) staining

After euthanasia under isoflurane anesthesia, ophthalmic scissors were used to separate the skin of the mouse's head. The scissors were gently inserted into the nostrils on both sides, the back of the nose was cut, the nasal bone was lifted, and the nasal septum and nasal cavity were exposed. The nasal septum was separated and fixed in a 10% neutral formalin solution. Four-micrometer-thick white sections were prepared after embedding and sectioning, and then stained with HE. After HE staining, the average value of eosinophils in three different high-power fields (HPF, 400 ×) was counted as the number of eosinophils infiltrated in the nasal septum of each mouse. Two independent investigators who were blinded to the study reviewed all slide images.

### Enzyme-linked immunosorbent assay (ELISA)

Blood samples were collected from anesthetized mice, placed at room temperature for 2 h, and centrifuged at 4000 r/min for 10 min at 4 °C to obtain mouse serum. The concentrations of interleukin (IL)-4, IL-5 and IFN-γ in serum were detected in accordance with the manufacturer’s instructions (MultiSciences, Hangzhou, China).

### Western blot

After the nasal septum was separated, the remaining nasal mucosa was scraped for Western blotting. The nasal mucosa was cut into small pieces and homogenized at a low temperature using an ultrasonic homogenizer. Total protein was extracted using RIPA buffer and isolated via 10% SDS-PAGE. The separated proteins were transferred to 0.22-μm PVDF membranes, which were incubated with primary antibodies (anti-CCR3, Affinity; anti-AKT, CST; anti-P-AKT, CST; and anti-GAPDH, Proteintech Group) at 4 °C overnight followed by incubation with the secondary antibody for 2 h.

### Statistical analysis

The data for each group are expressed as the means ± SEM and were processed using GraphPad Prism software (version 8.0, San Diego, CA, USA). One-way analysis of variance (ANOVA) was used for analyses. A *P* value < 0.05 was considered significant.

## Supplementary Information

Supplementary Information.

## Data Availability

All data generated or analysed during this study are included in this article (and its Supplementary Information files).

## References

[1] Bousquet J (2020). Allergic rhinitis. Nat. Rev. Dis. Primers.

[2] Meng Y, Wang C, Zhang L (2020). Advances and novel developments in allergic rhinitis. Allergy.

[3] Eifan AO, Durham SR (2016). Pathogenesis of rhinitis. Clin. Exp. Allergy.

[4] Greiner AN, Hellings PW, Rotiroti G, Scadding GK (2011). Allergic rhinitis. Lancet.

[5] Tang S, Shu X (2020). Effect of CCR3 gene on related inflammatory cells in respiratory allergic diseases. Lin Chung Er Bi Yan Hou Tou Jing Wai Ke Za Zhi.

[6] Grozdanovic M (2019). Novel peptide nanoparticle-biased antagonist of CCR3 blocks eosinophil recruitment and airway hyperresponsiveness. J. Allergy Clin. Immunol..

[7] Ahmadi Z, Hassanshahi G, Khorramdelazad H, Zainodini N, Koochakzadeh L (2016). An overlook to the characteristics and roles played by eotaxin network in the pathophysiology of food allergies: allergic asthma and atopic dermatitis. Inflammation.

[8] Salter BM, Ju X, Sehmi R (2021). Eosinophil lineage-committed progenitors as a therapeutic target for asthma. Cells.

[9] Fulkerson PC (2006). A central regulatory role for eosinophils and the eotaxin/CCR3 axis in chronic experimental allergic airway inflammation. Proc. Natl. Acad. Sci. U.S.A..

[10] Watanabe S, Yamada Y, Murakami H (2020). Expression of Th1/Th2 cell-related chemokine receptors on CD4(+) lymphocytes under physiological conditions. Int. J. Lab. Hematol..

[11] Zhu XH, Liao B, Liu K, Liu YH (2014). Effect of RNA interference therapy on the mice eosinophils CCR3 gene and granule protein in the murine model of allergic rhinitis. Asian Pac. J. Trop. Med..

[12] Wu S (2020). Effects of lentivirus-mediated CCR3 RNA interference on the function of mast cells of allergic rhinitis in mice. Int. Immunopharmacol..

[13] Vyas P, Vohora D (2017). Phosphoinositide-3-kinases as the novel therapeutic targets for the inflammatory diseases: Current and future perspectives. Curr. Drug Targets.

[14] Sotsios Y, Ward SG (2000). Phosphoinositide 3-kinase: A key biochemical signal for cell migration in response to chemokines. Immunol. Rev..

[15] Kang BN (2012). The P110delta subunit of PI3K regulates bone marrow-derived eosinophil trafficking and airway eosinophilia in allergen-challenged mice. Am. J. Physiol. Lung Cell. Mol. Physiol..

[16] Huang L (2018). OX40L induces helper T cell differentiation during cell immunity of asthma through PI3K/AKT and P38 MAPK signaling pathway. J. Transl. Med..

[17] Park SJ (2010). Phosphoinositide 3-kinase delta inhibitor suppresses interleukin-17 expression in a murine asthma model. Eur. Respir. J..

[18] Campa CC (2018). Inhalation of the prodrug PI3K inhibitor CL27c improves lung function in asthma and fibrosis. Nat. Commun..

[19] Shamri R, Young KM, Weller PF (2013). PI3K, ERK, P38 MAPK and integrins regulate CCR3-mediated secretion of mouse and human eosinophil-associated RNases. Allergy.

[20] Saito Y (2014). The effect of pharmacological PI3Kgamma inhibitor on eotaxin-induced human eosinophil functions. Pulm. Pharmacol. Ther..

[21] Hutchings CJ (2020). A review of antibody-based therapeutics targeting G protein-coupled receptors: An update. Expert Opin. Biol. Ther..

[22] Anderson CA (2020). A degradatory fate for CCR4 suggests a primary role in Th2 inflammation. J. Leukoc. Biol..

[23] Tang H, Li T, Han X, Sun J (2019). TLR4 antagonist ameliorates combined allergic rhinitis and asthma syndrome (CARAS) by reducing inflammatory monocytes infiltration in mice model. Int. Immunopharmacol..

[24] Kos CH (2004). Cre/loxP system for generating tissue-specific knockout mouse models. Nutr. Rev..

[25] Nagakubo D, Yoshie O, Hirata T (2016). Upregulated CCL28 expression in the nasal mucosa in experimental allergic rhinitis: Implication for CD4(+) memory T cell recruitment. Cell. Immunol..

[26] Peng H (2020). CCR3-shRNA promotes apoptosis and inhibits chemotaxis and degranulation of mouse mast cells. Exp. Ther. Med..

[27] Wang Y, Wang W, Wang L, Wang X, Xia J (2012). Regulatory mechanisms of interleukin-8 production induced by tumour necrosis factor-alpha in human hepatocellular carcinoma cells. J. Cell. Mol. Med..

[28] Ebrahimi S (2017). Targeting the Akt/PI3K signaling pathway as a potential therapeutic strategy for the treatment of pancreatic cancer. Curr. Med. Chem..

[29] Huang P (2017). Comprehensive attenuation of IL-25-induced airway hyperresponsiveness, inflammation and remodelling by the PI3K inhibitor LY294002. Respirology.

[30] Feng S (2018). Role of the TSLP-DC-OX40L pathway in asthma pathogenesis and airway inflammation in mice. Biochem. Cell Biol..

[31] Wang W (2012). Interleukin-25 promotes basic fibroblast growth factor expression by human endothelial cells through interaction with IL-17RB, but not IL-17RA. Clin. Exp. Allergy.

[32] Saw S, Arora N (2016). PI3K and ERK1/2 kinase inhibition potentiate protease inhibitor to attenuate allergen induced Th2 immune response in mouse. Eur. J. Pharmacol..

[33] Mendoza MC, Er EE, Blenis J (2011). The Ras-ERK and PI3K-mTOR pathways: Cross-talk and compensation. Trends Biochem. Sci..

[34] Yang H (2017). Brain-specific SNAP-25 deletion leads to elevated extracellular glutamate level and schizophrenia-like behavior in mice. Neural Plast..

[35] Niu Y (2020). HIF1alpha deficiency in dendritic cells attenuates symptoms and inflammatory indicators of allergic rhinitis in a SIRT1-dependent manner. Int. Arch. Allergy Immunol..

