# Supplementary material for: Gene knockdown of CCR3 reduces eosinophilic inflammation and the Th2 immune response by inhibiting the PI3K/AKT pathway in allergic rhinitis mice
Source: Sci Rep. 2022 Mar 30;12:5411. doi: 10.1038/s41598-022-09467-4 (PMC8969185; doi:10.1038/s41598-022-09467-4)

SUPPLEMENTARY INFORMATION

Gene knockdown of CCR3 reduces eosinophilic inflammation and the Th2 immune response by inhibiting the PI3K/AKT pathway in allergic rhinitis mice

Jiasheng Yuan, Yuehui Liu, Juan Yu, Meina Dai, Yu Zhu, Youwei Bao, Haisen Peng, Ke Liu, Xinhua Zhu*

Department of Otolaryngology-Head and Neck Surgery, The Second Affiliated Hospital of Nanchang University, Nanchang, 330006, China

*Corresponding Author: Prof. Xinhua Zhu, email: zhuxinhua2003@126.com

Supplementary Figure S1

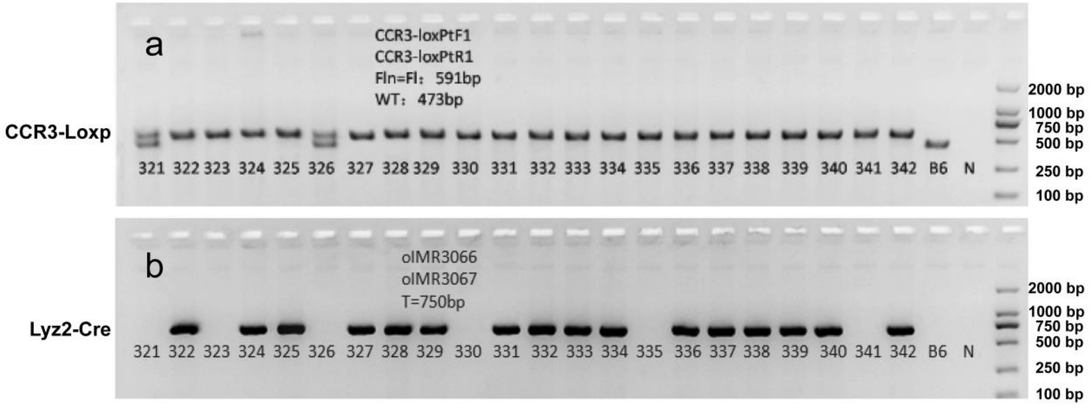

Supplementary Figure S1. The PCR products were detected by agarose gel electrophoresis for gene identification of progeny. (a) The CCR3-Loxp gene (591 bp), (b) the Lyz2-Cre gene (750 bp). The mice containing both CCR3-Loxp and Lyz2-cre genes were identified as CCR3$^{-/-}$ (CKO) mice. B6: negative control; N: No-Template Control.

Supplementary Table S1

Score	Nasal rubbing	Sneezing	Running nose
0	Without	Without	Without
1	Sporadically	Less than 3 times	To the anterior nostril
2	Repeatedly	More than 3 times but less than 10 times	Beyond the anterior nostril
3	Continuously	More than 11 times	All over the face

Supplementary Table S1. The scoring scale of nasal rubbing, sneezing and running nose of mice within 10 min after the final challenge.

Original western blot for three repeats

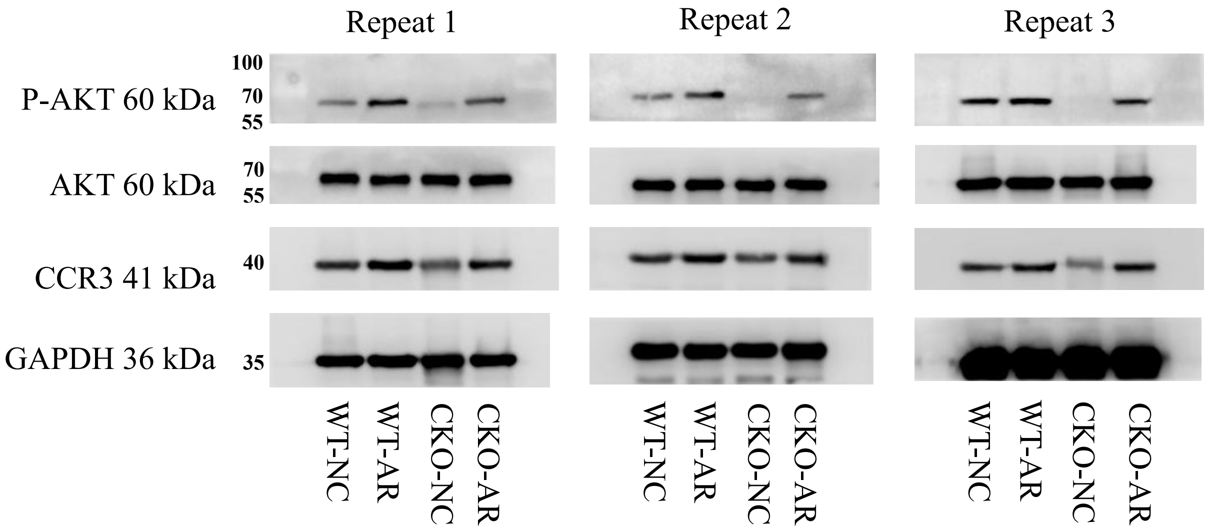

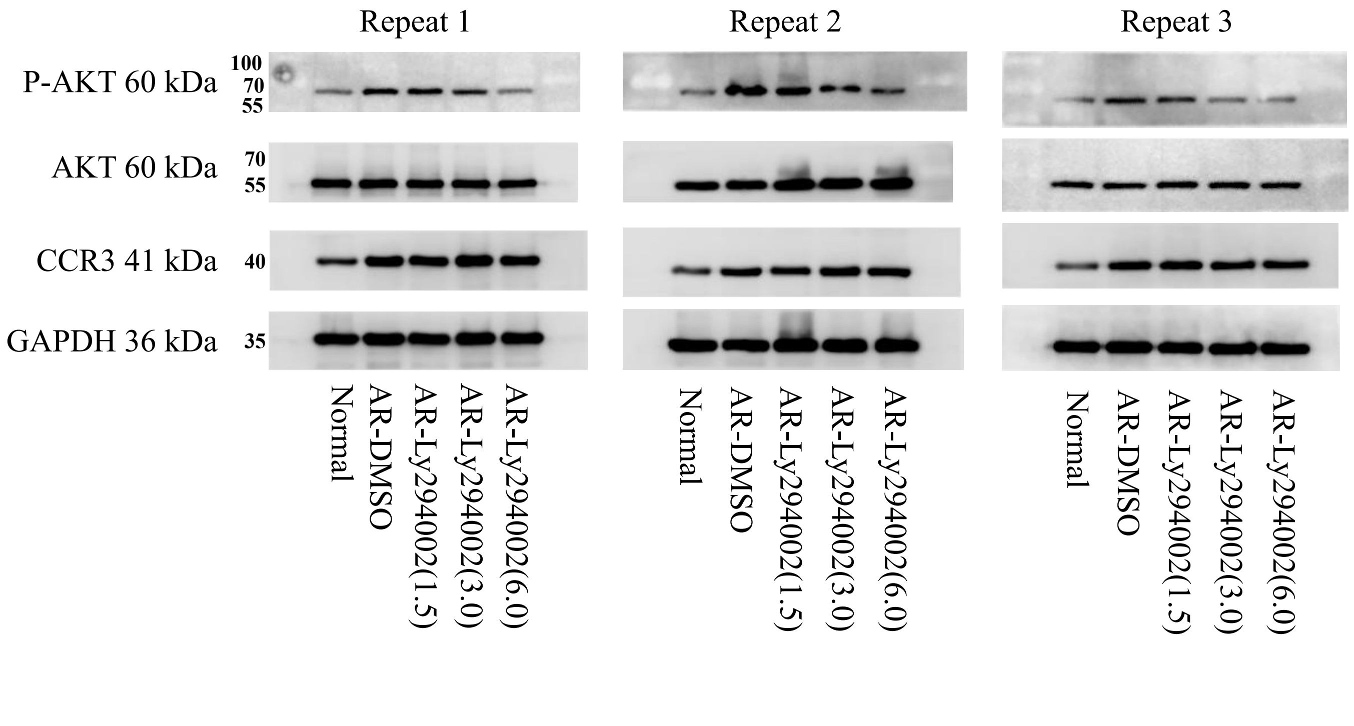

Supplement: Supplementary file 1 — Supplementary Information. [file 41598_2022_9467_MOESM1_ESM.pdf]
